# Peroxisome Proliferators-Activated Receptor (PPAR) Modulators and Metabolic Disorders

**DOI:** 10.1155/2008/679137

**Published:** 2008-06-15

**Authors:** Min-Chul Cho, Kyoung Lee, Sang-Gi Paik, Do-Young Yoon

**Affiliations:** ^1^Department of Bioscience & Biotechnology, Konkuk University, Seoul 143-701, South Kore; ^2^Department of Microbiology, Changwon National University, Changwon 641-773, South Korea; ^3^Department of Biology, Chungnam National University, Daejeon 305-764, South Korea

## Abstract

Overweight and obesity lead to an increased risk for metabolic disorders such as impaired glucose regulation/insulin resistance, dyslipidemia, and hypertension. Several molecular drug targets with potential to prevent or treat metabolic disorders have been revealed. Interestingly, the activation of peroxisome proliferator-activated receptor (PPAR), which belongs to the nuclear receptor superfamily, has many beneficial clinical effects. PPAR directly modulates gene expression by binding to a specific ligand. All PPAR subtypes (*α*, *γ*, and *σ*) are involved in glucose metabolism, lipid metabolism, and energy balance. PPAR agonists play an important role in therapeutic aspects of metabolic disorders. However, undesired effects of the existing PPAR agonists have been reported. A great deal of recent research has focused on the discovery of new PPAR modulators with more beneficial effects and more safety without producing undesired side effects. Herein, we briefly review the roles of PPAR in metabolic disorders, the effects of PPAR modulators in metabolic disorders, and the technologies with which to discover new PPAR modulators.

## 1. OBESITY, ADIPOCYTES, AND ADIPOKINES

Obesity, which is defined as excess adiposity for a given body size, results from an imbalance between energy intake and energy expenditure. Body mass index (BMI), measured as body weight in kilograms over the square of the height in meters (kg/m^2^), represents a widely accepted measure of adiposity. Wealth in industrialized societies, combined with an often-sedentary lifestyle and plentiful, high-calorie diets, creates irreversible weight gain. This social phenomenon can adversely impact well-being. Due to explosive concern for health and well-being, genes associated with human obesity are currently being defined, and whole genome scans will soon unveil its underlying genetic loci. The various causes of obesity are grouped according to behavioral (activity levels, nutrition, smoking status, and socioeconomic status), metabolic (physiological endocrine factors), and biological (genetic, racial, gender, age, and pregnancy status) influences [1]. Obesity has been recognized as a chronic disease since the National Institutes of Health Consensus conference in 1985 [2]. The increase in the prevalence of obesity has led the World Health Organization (WHO) to recently refer to the obesity issue as a “global epidemic”.

**Figure 1:**
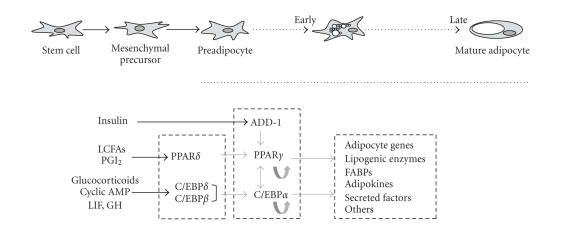
Adipocyte differentiation (adipogenesis) and transcriptional events in adipogenesis. A pluripotent stem cell precursor gives rise to a multipotential mesenchymal precursor cell with a potential to differentiate into an adipocyte. The preadipocyte enters the adipogenesis stage via environmental and gene expression signals. In an early stage of adipogenesis, major transcriptional factors such as PPAR*γ* and C/EBP*α* are expressed, and these factors strongly regulate the expressions of adipogenesis-related genes. The adipocyte secretes various factors, including adipokines, and the secreted factors play an important role in glucose and lipid metabolism, immune system, appetite regulation, and vascular disease. LCFAs: long-chain fatty acids; PGI_2_: prostacyclin; LIF: leukemia inhibitory factor; GH: growth hormone; ADD-1: adipocyte determination and differentiation factor-1; FABPs: fatty acid-binding proteins.

Chronic disruption of the energy balance due to exceeding energy intake causes hypertrophy and hyperplasia of fat cells, and this is representative of the pathology of obesity. When the intake of energy chronically exceeds energy expenditure, most of the excess energy is stored in the form of triglyceride in adipose tissue (from Greek *adip*- or *adipo*, mean fat). Increased adipose tissue mass can arise through an increase in cell size, cell number, or both. Adipocytes are remarkably variable in size, which reflects the amount of stored triglyceride. Mild obesity mainly reflects an increased adipose cell size (hypertrophic obesity), while more severe obesity or obesity arising in childhood typically also involves an increased number of fat cells (hyperplastic obesity) [3]. As a key part of the homeostatic system that controls energy balance, the molecular mechanisms that regulate preadipose cell growth (proliferation), adipose differentiation (adipogenesis), and lipogenesis have been subject to extensive scrutiny. An overview of cell types and molecular events that occur during adipogenesis is presented in Figure 1. Preadipocytes undergo growth arrest, postconfluent mitosis, and clonal expansion following appropriate environmental and gene expression cues. The committed preadipocytes must then withdraw from the cell cycle before adipose conversion. During the differentiation of adipocytes, the adipocyte phenotypes are characterized by sequential changes in the expression of numerous genes [4, 5]. The study of the cellular and molecular events of adipogenesis was facilitated by the establishment of preadipose cell lines. Among these cell lines, some are derived from embryonic cells such as the 3T3-L1 and 3T3-F442A cell lines, and others such as the Ob17 cell line and its subclones, which originated from adult animals [6]. When maintained in appropriate culture conditions, these cells undergo an adipose conversion characterized by the transcriptional activation of numerous genes. The process of adipose conversion is controlled by external signals, and it has been found that adipogenic cocktails are different depending on the cell systems used. For instance, the 3T3-L1 preadipose cells are induced to differentiate by a treatment with high concentrations of cyclic AMP, dexamethasone, and insulin at the preadipose stage. Hormones such as insulin, triiodothyronine, glucocorticoides, and growth hormone exert positive actions on the differentiation of adipose cells. Prostaglandins such as prostacyclin (PGI_2_), prostaglandin D_2_, and 15-deoxy-Δ^12, 14^-PGJ_2_ (15d-PGJ_2_) have also been found to be strong activators of adipogenesis. Several transcription factors have been shown to act cooperatively and sequentially to control adipogenesis. These include members of the transcription factors, such as CCAAT/enhancer-binding protein *α* **(C/EBP*α*) [7], peroxisome proliferator-activated receptor-*γ* (PPAR-*γ*), and adipocyte determination, as well as differentiation factor-1 (ADD-1). The last stage of terminal differentiation corresponds to the activation of several genes, including those for proteins involved in triglyceride metabolism [8].

Adipose tissue is partitioned into a few large depots (subcutaneous and visceral locations), and many small depots (heart, epicardium, pericardium, large blood vessels, major lymph nodes, bone marrow, kidney, adrenal glands, and the brain) [9]. All adipocytes secrete a large number of multifunctional molecules, including cytokines, growth factors, enzymes, hormones, complement factors, matrix proteins, and so forth. The proteins that are secreted from adipocytes are designated “adipokines” or “adipocytokines”. Since the isolation of the first-known adipocyte-secreted protein (the serine protease adipsin) in 1987 [10], the list of adipokines has been greatly extended. Leptin (from Greek *leptos*, means thin), encoded by the obese (*ob*) gene [6, 11], adiponectin (also called Acrp30) [12, 13], Interleukin-6 (IL-6), tumor necrosis factor-*α* (TNF-*α*) [14], resistin [15], and visfatin [16] are candidates of great interest among the growing number of factors found to be secreted by adipocytes. It has recently been shown that adipokines that are secreted from adipocytes contribute to the development of obesity-associated metabolic disorders, including insulin resistance, cardiovascular disease, and cancer [17].

## 2. METABOLIC DISORDERS AND THERAPEUTIC TARGETS

Overweight and obesity lead to increased risk for noninsulin-dependent diabetes, hypertension, coronary artery disease, dyslipidemia, gallstones, osteoarthritis, sleep apnea, certain forms of cancer, and degenerative arthritis. As the prevalence of obesity has increased, the heterogeneous clinical disorder strongly associated with abdominal obesity and insulin resistance has been identified as a major risk factor for atherosclerotic cardiovascular disease. This disorder, previously termed “syndrome X” by Reaven, and “insulin-resistance syndrome” by others, is now considered to be metabolic syndrome or metabolic disorder [18]. This disorder shares similar cardiovascular risk factors, including abdominal obesity, impaired glucose regulation/insulin resistance, dyslipidemia, and hypertension. Accordingly, these factors define the clustering of findings typical of the metabolic disorders, and establish diagnostic criteria. A number of studies have shown that the excess body fat that is stored in the deep abdominal area is associated with metabolic complications [19]. Recently, several molecular drug targets with potential to prevent or treat metabolic disorders have been revealed.

The excess glucocorticoid action by the enzyme 11*β*-HSD (hydroxysteroid dehydrogenase) type 1 induces obesity and features of metabolic disorders. Transgenic mice which are selectively over-expressing 11*β*-HSD1 in adipose tissue lead to increased food intake and body weight, as well as the development of visceral obesity. In addition, insulin-resistant diabetes, hyperlipidemia, and hyperphagia were observed in 11*β*-HSD1 transgenic mice [20]. On the other hand, 11*β*-HSD1 deficiency causes favorably altered fat distribution and adipose insulin sensitization. Even with high-fat and cholesterogenic diets, lipid profiles are also improved [21]. 11*β*-HSD1 inhibitors might have beneficial consequences in metabolic disorder. For instance, carbenoxolone, an 11*β*-HSD1 inhibitor, reduced total cholesterol in healthy subjects, and decreased the glucose production rate during hyperglucagonemia in diabetic patients [22]. AMP-activated protein kinase (AMPK) is a major regulator of lipid and glucose metabolism, and AMPK activation appears as a benefit of exercise in diabetic patients. Activation of AMPK by metformin decreased the level of plasma glucose and plasma triglycerides by promoting muscle glucose uptake and inhibiting hepatic glucose output [23]. SCD-1 (stearoyl CoA desaturase-1) is required for the biosynthesis of the monounsaturated fatty acids from saturated fatty acids, and SCD-1-deficient mice appear visibly lean compared to their littermates. SCD-1 deficiency in *ob/ob* mice ameliorates obesity and completely corrects the excessive hepatic lipid storage and VLDL production of the hypometabolic phenotype in leptin deficiency [24]. An SCD-1 inhibitor that reduces SCD-1 activity may serve as a therapeutic strategy for metabolic disorders, but very few reports are available for the use of SCD-1 inhibitor. I*k*B kinase *β* (IKK*β*) plays a key role in the activation of NF-*k*B by phosphorylating I*k*B*α*. It has recently been reported to act as a key role in obesity-linked insulin resistance. In obese rodents, increased IKK activity or overexpressed IKK promotes insulin resistance, whereas reduction of IKK activity or IKK*β* expression improves insulin sensitivity. In addition, high doses of IKK*β* inhibitors such as aspirin and salicylate reverse insulin resistance by sensitizing insulin signaling in obese rodents [25]. Protein tyrosine phosphatase 1B (PTP1B) is closely associated with insulin signaling through the dephosphorylation of activated insulin receptor or insulin receptor substrates. PTP1B deficiency and its heterozygote significantly reduce glucose concentrations in the blood, and PTP1B deficiency causes a significant reduction of circulating insulin concentration compared to wild-type mice. When on a high-fat diet, PTP1B-deficient mice were resistant to diet-induced weight gain, and remained insulin-sensitive [26]. Because PTP1B inhibition provides attractive therapies against metabolic disorders, various studies for the inhibition mechanism of inhibitors against PTP1B, the structure-activity relationship, and synthetic and pharmacological materials have been performed by different groups. Acetyl-CoA carboxylase (ACC) is a key determinant of energy homeostasis because increased malonyl-CoA by ACC activation inhibits mitochondrial fatty acid uptake and oxidation. A lack of malonyl-CoA in the muscle and heart of ACC2-deficient mice show increased oxidation of fatty acid, decreased fat in adipose and liver tissue, and decreased the storage of glycogen in the liver [27]. CP-640186, an isozyme-nonselective ACC inhibitor, inhibits fatty acid and TG synthesis in HepG2 cells, as well as fatty acid synthesis in obese rodents. CP-640186 also stimulates fatty acid oxidation in C2C12 cells [28]. These effects of the ACC inhibitor may provide novel therapeutic potential for treatment of the metabolic disorder. Interestingly, the activation of PPARs by their ligands has many beneficial effects in the improvement of glucose homeostasis and lipid homeostasis.

## 3. PPARs AND METABOLIC DISORDERS

### 3.1. PPARs as a nuclear receptor family

Peroxisomes are subcellular organelles that perform diverse metabolic functions, including H_2_O_2_-derived respiration, *β*-oxidation of fatty acids, and cholesterol metabolism. Rodents exposed to peroxisome proliferators lead to hepatocellular hypertrophy, hyperplasia, and transcriptional induction of fatty acid-metabolizing enzymes that are regulated in parallel with peroxisome proliferation [29]. Peroxisome proliferators may activate PPARs by binding directly to the receptors, and the activated PPARs may regulate the expression of genes involved in lipid metabolism and peroxisome proliferation. Recent research on PPARs has moved toward their pivotal roles comprising one family of nuclear receptors [30]. Nuclear receptors, which are present in multicellular organisms, directly control the expression of genes in response to a wide range of developmental, physiological, and environmental signals.

The PPARs of nuclear receptors mainly consist of three subtypes (PPAR*α*, PPAR*γ*, and PPAR*δ*/*β*). All three PPAR isoforms possess similar structural and functional features. Principally, four functional domains have been identified, and are referred to as A/B, C, D, and E/F. The N-terminal A/B domain contains ligand-independent activation function 1 (AF-1). The ligand-independent activation region can confer constitutive activity on the receptor, and is negatively regulated by phosphorylation [31]. The DNA-binding domain (DBD) or C domain consists of two zinc fingers, and is directly involved with the binding of PPAR to the peroxisome proliferator response element (PPRE) in the promoter regions of target genes. PPREs are direct repeat (DR)-1 elements consisting of two hexanucleotides with the AGGTCA consensus sequence separated by a single nucleotide spacer. Such a sequence, or a similar one, has been found in numerous PPAR-inducible genes, including acyl-CoA oxidase (ACO) and adipocyte fatty acid-binding protein (aP2) [32]. The D site is a hinge region and a docking domain for corepressors. The E/F domain or ligand-binding domain (LBD) is responsible for ligand specificity and the activation of PPAR binding to the PPRE, which increases the expression of the targeted gene. Upon the binding of a specific ligand to LBD of the E/F domain, the conformation of a PPAR is altered and stabilized. The ligand-bound LBD results in the recruitment of transcriptional coactivators, resulting in gene transcription. Although three of the PPAR isoforms possess similar structures, it is clear that these receptors perform distinct functions according to the specific ligands and their expression patterns in the tissues.

### 3.2. PPARs and their ligands

**Figure 2:**
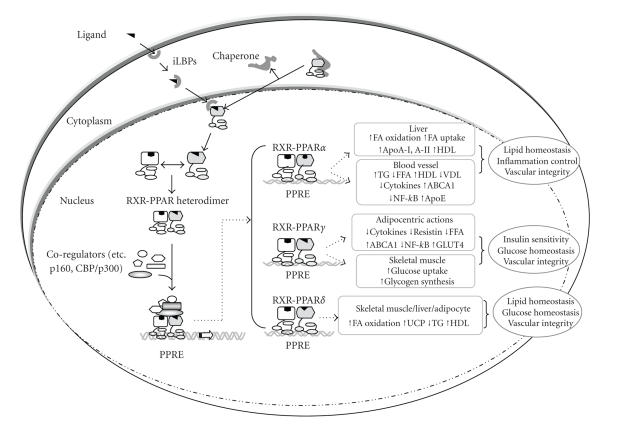
Signaling pathways activating PPAR and regulating the biological effects of PPAR in different organs. PPAR activity can be regulated by the direct binding of small lipophilic ligands. Ligand-unbound PPAR in the cytosol is associated with chaperons, and the association changes the conformation of PPAR that allows for high-affinity binding to the ligand. Ligand-bound PPAR forms a heterodimer with RXR, and the PPAR-RXR heterodimer constructs the transcriptional machinery through the recruitment of coregulators. The transcriptional machinery regulates gene expression by binding to specific DNA sequence elements, termed PPAR response elements (PPRE). PPAR*α* is strongly expressed in the liver, heart, and blood vessels, and regulates the expressions of genes related to lipid metabolism and inflammation control. PPAR*γ* exerts its effects on insulin sensitivity and glucose homeostasis in adipocytes and skeletal muscles. PPAR*δ* is expressed ubiquitously, and controls the expressions of genes that are involved in glucose and lipid metabolism. FA: fatty acid; HDL: high-density lipoprotein; VDL: very low-density lipoprotein; ABCA1: ATP-binding cassette transporter A1; UCP: uncoupling protein; TG: triglyceride.

Ligand-induced activation of the PPAR, by means of low-affinity binding to natural lipid ligands, stimulates an array of molecular responses that aim at maintaining lipid and glucose homeostasis. Ligand-unbound PPAR is associated with chaperon in the cytosol, and the association induces the PPAR to be held in a conformation that allows for high-affinity binding of the ligand [33]. The translocation of a hydrophobic ligand into the cell is facilitated in intra- and extracellular fluids by intracellular lipid-binding proteins (iLBPs) that are members of the family of fatty acid-binding proteins (FABPs). The iLBPs with relatively small sizes (15-16kDa) play important roles in the solubilization and protection of ligands in aqueous spaces. Ligand-loaded iLBP in the cytosol translocates into the nucleus by free diffusion, and they form a short-lived complex with PPAR [34–36]. Ligand is then transferred to the PPAR, and the ligand-bound PPAR forms a heterodimer with the partner nuclear receptor, retinoid X receptor (RXR*α*). Upon binding to a ligand, the conformation of PPAR is altered and stabilized, and the PPAR-RXR heterodimer then recruits transcriptional coactivators [37–39]. The transcription machinery is bound to PPRE, and directly controls the expression of the target gene (Figure 2) [40].

PPAR*α* was first cloned from the rodent liver in 1990 [41], and PPAR*β* and PPAR*γ* were first identified in *Xenopus* [42]. Several groups subsequently reported the cloning of mammalian orthologs of PPAR*α*, PPAR*β*, and PPAR*γ*. Although PPAR*α* and PPAR*γ* are highly conserved across species, PPAR*β* varies considerably between *Xenopus* and mammals. The murine clone was named PPAR*δ* because of this divergence [43]. PPAR*α* is predominantly expressed in the liver, and is involved in peroxisome proliferation and regulation of fatty acid catabolism. The expression of PPAR*δ* is ubiquitous and abundant in the brain, intestine, skeletal muscle, spleen, macrophages, lung, and adrenals [44]. PPAR*δ* is activated by a large variety of ligands, and has been implicated in developmental and metabolic regulation in several tissues. PPAR*γ* is expressed in adipose tissue, promoting adipogenesis and increasing lipid storage. PPAR*γ* has at least two promoters, and results in the production of two isoforms, 1 and 2. These isoforms are expressed in a tissue-specific pattern. The PPAR*γ*1 isoform is expressed in the spleen, intestine, and white adipose tissue, while the PPAR*γ*2 is preferentially expressed in white and brown fat. PPAR*γ*2 is most abundantly expressed in fat cells, and plays a pivotal role in fat cell differentiation and lipid storage [45]. The distinct physiological roles of each subtype have been shown to be determined by binding to a discrete set of ligands. Although fatty acids could activate PPARs, PPAR*α* activity was induced by eicosanoids [46], cabaprostacyclin [47], and nonsteroidal anti-inflammatory drugs (NSAIDs) [48]. PPAR*δ* was activated by several polyunsaturated fatty acids [49] and eicosanoids [50]. PPAR*γ* specifically binds to thiazolidinediones (TZDs), a class of antidiabetic drugs. Other PPAR*γ* ligands include the natural prostaglandin metabolite 15-deoxy-Δ^12.14^-prostaglandin J_2_(PGJ_2_), polyunsaturated fatty acids, and NSAIDs such as ibuprofen and indomethacin [51, 52].

### 3.3. Post-translational regulation of PPARs

PPARs and other nuclear receptors modulate their transcriptional activity via phosphorylation by various kinases, including the mitogen-activated protein kinase (ERK MAPK and p38 MAPK), protein kinase A and C (PKA and PKC), AMP kinase (AMPK), and glycogen synthase kinase 3 (GSK3) [53]. Several mechanisms have been described to explain the modulation of PPAR transcriptional function. First, phosphorylation modulates the affinity of PPARs for their ligand, as well as the coactivator recruitment abilities of PPARs. Although the main phosphorylation site of PPAR*γ* (Ser 112) is located far from the ligand-binding domain, mutated PPAR*γ* (S112D) exhibits a decreased ligand-binding affinity and decreased coactivator recruitment [54]. Second, the phosphorylation of PPARs modulates binding to PPRE. In gel retardation experiments, PPAR*α* phosphorylation via PKA enhances gene expression due to the stabilization of the binding of PPAR*α* to DNA [55]. Finally, phosphorylation plays an important role in ubiquitination and proteasomal catabolism of PPARs. Phosphorylation of the PPAR*γ* AF-1 domain by IFN*γ*-ERK-regulated serine phosphorylation promotes the degradation of PPAR*γ* by the ubiqutin-proteasome-dependent degradation in response to ligand activation [56]. PPAR*α* is also degraded by the ubiquitin-proteasome-dependent degradation. However, in contrast to PPAR*γ*, phosphorylation of PPAR*α* induces the stabilization of PPAR*α* by reducing ubiquitination. The phosphorylation and interaction of PPAR*α* with a corepressor stabilize PPAR*α* protein by decreasing its ubiquitination in order to keep a pool of PPAR*α* available for ligand binding and activation [57].

SUMOylation consists of the covalent and reversible conjugation of small ubiquitin-related modifiers (SUMOs) to target protein and regulate biological processes. The number of known SUMO targets is growing, and SUMOylation of PPAR*γ* has recently been reported. SUMOylation of PPAR*γ* mainly occurs at a lysine residue within a ligand-independent activating function domain (AF-1). PPAR*γ* is SUMOylated by SUMO-1 and PIAS proteins that function as E3 ligases [58]. Potential SUMOylation sites of PPAR*γ* include K77 (equivalent to K107 of PPAR*γ*2) and K365. SUMOylation of PPAR*γ* at K77 and K365 occurs in a ligand-dependent manner. SUMOylation of K107 inhibits PPAR*γ*-dependent gene induction, but does not affect transrepression, whereas mutation of K365 eliminates the ability of agonist-activated PPAR*γ* to repress iNOS and to be recruited to its promoter [59]. Phosphorylation at S112 of PPAR*γ*2 promotes K107 SUMOylation and exerts more potent repressive effects. The SUMOylation-defective mutation of PPAR*γ* at K77R promotes adipocyte differentiation. The potential SUMOylation site of PPAR*α* has one K185 within the D region, and PPAR*δ*/*β* has one K104 in the C region, but in vivo SUMOylation is specific for PPAR*γ* among the PPARs [60]. Relatively few studies of post-translational regulation of PPARs have been reported.

### 3.4. Role of PPAR ligands in metabolic disorders

The activation of PPAR*α* upregulates the expressions of several catabolic enzymes that are involved in mitochondrial and peroxisomal *β*-oxidation and microsomal *ω*-oxidation, as well as in the transcriptional regulation of genes that are necessary for the maintenance of the redox balance during the oxidative catabolism of fatty acids. The derivatives from fatty acids and fibrates, including gemfibrozil, fenofibrate, cofibrate, bezafibrate, and ciprofibrate, can activate PPAR*α*. These fibrates are used in the treatment of hypertriglyceridemia. PPAR*α* agonists fundamentally regulate *β*-oxidation of fatty acids, and promote the expression of cytochrome P450 enzymes, which catalyze the *ω*-hydroxylation of fatty acid [61]. WY14,643, a well-known specific PPAR*α* agonist, increases fatty acid oxidation by increasing the expressions of peroxisomal and mitochondrial fatty acid *β*-oxidation enzymes. WY14,643 reduces liver insulin resistance more efficiently than muscle insulin resistance by normalizing the circulating triglyceride levels and blood glucose levels in lipoatrophic mice [62]. PPAR*α* agonists also activate the expression of apolipoprotein A-1 (ApoA-1) and ATP-binding cassette transporter A1 (ABCA1) [63, 64]. The increased ApoA-1 and ABCA1 proteins enhance cholesterol efflux by the reverse cholesterol transport (RCT) pathway. In addition, PPAR*α* agonists have anti-inflammatory effects in vascular cells. WY14,643 or bezafibrate induces PPAR*α*-mediated inhibition of osteopontin (OPN) expression in human macrophages of atherosclerotic lesions, where they are abundantly synthesized. Bezafibrate significantly decreases OPN plasma levels in type 2 diabetic patients [65]. Therefore, the PPAR*α* agonist reduces the progression of atherosclerosis and decreases the incidence of coronary heart disease [66]. However, fibrates are contraindicated in patients with renal insufficiency, gallstones, abnormal liver function tests, and pregnancy [67].

The activation of PPAR*γ* promotes the storage of fat by increasing adipocyte differentiation and enhancing the transcription of genes that are important for lipogenesis. The activation of either PPAR*α* or PPAR*γ* in macrophages promotes the cellular efflux of phospholipids and cholesterol in the form of high-density lipoproteins by upregulating the expression of the liver X-receptor (LXR), an oxysterol-activated nuclear hormone receptor that increases expression of the lipid transporter ABCA1 (ATP-binding cassette, subfamily A, member 1) [68]. PPAR*γ* has been the focus of intense research during the past decade because ligands for this receptor have emerged as potent insulin sensitizers that can be used in the treatment of type 2 diabetes [69]. Increased levels of circulating free fatty acids and lipid accumulation in non-adipose tissue have been implicated in the development of insulin resistance. This situation is improved by the PPAR*γ* agonist, which promotes fatty acid storage in fat depots and regulates the expression of adipocyte-secreted hormones that impact glucose homeostasis [70]. The net result of the pleiotropic effects of the PPAR*γ* agonist is improvement of insulin sensitivity, although undesired side effects limit the utility of this therapy. In fact, TZD, a synthetic agonist of PPAR*γ*, appears to be ideally suited for the treatment of this cluster of metabolic abnormalities, which has been termed the insulin resistance or cardiovascular dysmetabolic syndrome as a whole [71]. Two compounds in this class are currently approved for use in the United States. They are Rosiglitazone (Avandia), approved by the US Food and Drug Administration (FDA) in May 1999, and Pioglitazone (Actos), which was approved in July 1999. Historically, the first agent in this class, Troglitazone (Rezulin), was marketed in the United States from March 1997 to March 2000. Troglitazone was banned because the FDA determined that the risk of idiosyncratic hepatoxicity associated with Troglitazone therapy outweighed its potential benefits [72, 73].

The activation of PPAR*δ* in macrophages also upregulates the expression of the ABCA1 transporter. Recent evidence indicates that PPAR*δ* can also promote cellular lipid accumulation by increasing the expressions of genes that are involved in lipid uptake, and by repressing key genes that are involved in lipid metabolism, inflammation, atherosclerosis, obesity, fertility, and cancer [74, 75]. Several 14- to 18-carbon saturated fatty acids as well as 16- to 20-carbon polyunsaturated fatty acids are screened as PPAR*δ*-binding chemicals in ligand screening and competition binding assays [50, 76, 77]. As physiological ligands of PPAR*δ*, these fatty acids or eicosanoids are unsettled. However, Chawla et al. hypothesized that PPAR*δ* acts as a lipid sensor, where fatty acids derived from very-low-density lipoprotein (VLDL) can activate PPAR*δ* [78]. A PPAR*δ*-specific agonist, GW501516, decreases plasma triglyceride levels in obese monkeys, raises high-density lipoprotein levels, and prompts the initiation of clinical trials to assess its efficacy in hyperlipidemic patients [79]. GW501516 also attenuates weight gain and insulin resistance in mice fed high-fat diets by increasing the expressions of genes that promote lipid catabolism and mitochondrial uncoupling in skeletal muscle, thereby increasing *β*-oxidation of the fatty acids in skeletal muscle [80]. PPAR*δ* agonists also have anti-inflammatory properties. The PPAR*δ* agonist inhibits LPS-inducible genes, such as COX-2 and iNOS in murine peritoneal macrophages [81]. These reports indicate that the PPAR*δ*-specific agonist is a potential therapeutic interest for the treatment of metabolic disorder.

## 4. VARIOUS STRATEGIES FOR SAFER PPAR MODULATORS

Each PPAR subtype regulates a distinct metabolic pathway, and the agonists of each of the PPAR subtypes have distinct effects with undesired side effects such as weight gain, hepatotoxicity, and heart failure. In the case of the TZD class as PPAR*γ* agonists, the major side effect is weight gain. A Pro12Ala substitution in PPAR*γ*2 decreases PPAR*γ* activity, BMI, and insulin resistance [82]. Because of these undesirable effects caused by PPAR*γ* agonists, new therapeutic solutions have been investigated in order to reduce their side effects. Various compounds have been reported to be PPAR antagonists, including Bisphenol A diglycidyl ether (BADGE), PD068235, LG100641, GW9662, SR-202, GW6741, and Compound A and B [83]. A potent selective PPAR*γ* antagonist, GW9662, does not recruit PPAR coactivators such as SRC-1 and p300, and it suppresses rosiglitazone-induced adipocyte differentiation in 3T3-L1 adipocytes. GW9662 prevents high-fat diet-induced obesity without affecting food intake, and has no effect on high-fat diet-induced glucose intolerance [84]. The phosphonophosphate SR-202, a PPAR*γ* antagonist, inhibits BRL 49653-mediated recruitment of SRC-1 and troglitazone-induced transcriptional activity. SR-202 inhibits PPAR*γ*-induced adipocyte differentiation of 3T3-L1 and prevents weight gain and adipose tissue deposition in mice given a standard diet or high-fat diet. In addition, SR-202 markedly reduces hyperglycemia and hyperinsulinemia in ob/ob mice [85]. A few PPAR*α* antagonists have been reported, but in vivo data have not been disclosed. Several PPAR*γ* antagonists may have therapeutic availability as antiobesity drugs. However, further studies of the molecular effects of PPAR*γ* antagonists are necessary.

The combination agonist strategy, which uses a combination of agonists, has been designed to activate each receptor subtype. In terms of its pharmacological aspects, this strategy may provide more efficacious effects and more safety for undesired side effects. The possible combinations are PPAR*α*/*γ* dual agonist, PPAR*γ*/*δ* dual agonist, PPAR*α*/*δ* dual agonist, and PPARpan (PPAR*α*/*γ*/*δ*) agonist. The initial combination agonist strategy was focused on the development of PPAR*α*/*γ* dual agonists. PPAR*γ* agonists such as rosiglitazone and pioglitazone provide undesired side effects of TZDs, including weight gain. By contrast, PPAR*α* agonists such as fibrate decreased adiposity through the stimulation of lipid oxidation. Dual PPAR*α*/*γ* stimulation with a combination of rosiglitazone and fenofibrate in type 2 diabetic patients effectively improved the atherogenic dyslipidemic profile, which plays a key role in the occurrence of cardiovascular mortality [86]. Applications of structurally various PPAR*α*/*γ* dual agonists have recently been reported. Among these dual PPAR*α*/*γ* agonists, compounds belonging to the glitazar class have been advanced to clinical development (Phases II and III). These PPAR*α*/*γ* dual agonists commonly reduce triglycerides and total cholesterol, increase HDL levels, and consequently improve insulin sensitivity. However, the use of a few PPAR*α*/*γ* dual agonists, including muraglitazar, tesaglitazar, ragaglitazar, farglitazar, TAK559, and KRP297, has been discontinued due to various safety liabilities compared to selective agonists. All glitazars significantly increase weight gain and edema, because of higher PPAR*γ* affinity than PPAR*α* affinity although their affinity for PPAR*α* is higher than fibrates. Muraglitazar increases cardiovascular risks, tesaglitazar impairs glomerular filteration rate, and some have carcinogenic effects in mice [87]. The safety liabilities may be the result of their imbalanced activities on PPAR*γ* and PPAR*α*. Therefore, the best solution would be to screen candidates with appropriate affinity for PPAR*α* and selective PPAR*γ*-modulating activity [88].

Both PPAR*γ* and PPAR*δ* play important roles in glucose and lipid metabolism. A PPAR*γ*/*δ* dual agonist with a properly controlled *γ*/*δ* ratio could attenuate undesired weight gain, improve insulin sensitivity, and stimulate fatty acid oxidation. The dual PPAR*γ*/*δ* agonist (R)-3-{2-ethyl-4-[3-(4-ethyl-2-pyridin-2-yl-phenoxy)-butoxy]-phenyl}-propionic acid has been shown to lower the glucose level and cause less weight gain than rosiglitazone in hyperglycemic male Zucker diabetic fatty (ZDF) rats [89]. The other dual PPAR*γ*/*δ* agonist, (R)-3-{4-[3-(4-chloro-2-phenoxy-phenoxy)-butoxy]-2-ethyl-phenyl}-propionic acid, improves insulin sensitivity and reverses diabetic hyperglycemia with less weight gain relative to rosiglitazone in female ZDF rats [90]. PPAR*α*/*δ* dual agonists (T659 and Compound 24) have recently been reported. T659 has had beneficial effects on HDL-C in experimental primates [91]. Compound 24 has also shown significant effects on HDL-C, TG, and FFA levels in male hApoA1 transgenic mice [92]. PPAR*α*/*δ* dual agonists may improve hyperlipidemia, insulin resistance, and risk of atherosclerosis. The development of PPAR*α*/*δ* and PPAR*γ*/*δ* dual agonists is currently being pursued.

Another strategy to reduce the adverse effects of previous PPAR*γ* agonists is the identification of partial agonists, also referred to as selective PPAR*γ* modulators (SPPAR*γ*Ms). SPPAR*γ*Ms are PPAR*γ* ligands with insulin-sensitizing activity and lower stimulation of adipogenesis. Because SPPAR*γ*Ms bind to the ligand-binding pocket of the PPAR*γ* receptor in distinct manners, SPPAR*γ*M-bound PPAR*γ* induces the displacement of the differential cofactor and the specific gene expression in a tissue-specific manner. Although several PPAR*γ* agonists have been classified as SPPAR*γ*Ms, the majority of these synthetic ligands remain to be characterized at the molecular level, and need to be evaluated in in vivopreclinical models to assess their relationships with weight gain [93]. Halofenate (HA) and PA-082, new SPPAR*γ*Ms, were recently developed. HA causes displacement of corepressors (N-CoR and SMRT), but does not cause efficient recruitment of coactivators (p300, CBP, and TRAP 220). Moreover, HA selectively regulates the expression of multiple PPAR*γ* responsive genes in 3T3-L1 adipocytes, and has acute antidiabetic properties in diabetic *ob/ob* mice [94]. The isoquinoline derivative PA-082, a prototype of a novel class of non-TZD partial PPAR*γ* agonists, causes preferential recruitment of PPAR*γ*-coactivator-1*α* (PGC1*α*) to the receptor compared with rosiglitazone. PA-082 antagonizes rosiglitazone-driven transactivation and TG accumulation in C2H10T1/2 mesenchymal stem cells. However, PA-082 induces mRNAs of genes that encode components of insulin signaling pathways. It also facilitates glucose uptake and insulin signaling in mature adipocytes [95]. The functional study of SPPAR*γ*Ms will provide more information about effective antidiabetic agents to reduce the side effects of weight gain.

The PPARpan agonists can activate all three PPAR subtypes, and they can potentially exert various effects on metabolic disorders such as insulin resistance, obesity, dyslipidemia, and hypertension. The well-known lipid-lowering bezafibrate is the first clinically-tested PPARpan agonist. Though bezafibrate is a PPAR ligand with a relatively low potency, it considerably raises HDL cholesterol, reduces triglycerides, improves insulin sensitivity, and reduces blood glucose levels [96]. GW677954, a novel PPARpan agonist, is being investigated in Phase II trials for the treatment of metabolic disorders [97]. PLX-204 and GW-625019 are also progressing in Phase I trials for the treatment of metabolic disorders. In addition, LY-465608, DRF-11605, CS-204, and DRL-11605 are under investigation, and may be potent therapeutic agents for the treatment of metabolic disorders [88].

## 5. TECHNOLOGIES TO DISCOVER NEW PPAR MODULATORS

The development of new technology to discover PPAR modulator is significant in functional study of the nuclear receptors and new potent drug discovery. In general, transactivation and chimeric receptor transactivation assays have been used as cell-based methods employing mammalian cells for the screening of new PPAR modulators. Cell-based assays provide a more physiological relevance, but these assays are costly, time-consuming, and difficult to apply to automated systems used for high-throughput screening (HTS). Recently, Chen et al. introduced a yeast-based method for screening PPAR modulators [98]. Cell-free assays for the screening of PPAR modulators have been developed in numerous forms. The X-ray crystal structure study revealed that the human apo-PPAR*γ* ligand-binding domain (LBD) has a large binding pocket, which may explain the diversity of the PPAR*γ* ligands [99]. When binding to specific ligands in LBD, PPAR changes its conformation. Glutamate and lysine residues that are highly conserved in LBDs of PPAR form a “charge clamp” that contacts the backbone atoms of the LXXLL helices of coactivators such as steroid receptor coactivator-1 (SRC-1). In the case of SRC-1, four consecutive LXXLL motifs make identical contacts with both subunits of a PPAR-RXR heterodimer [100]. Such allosteric conformational changes promote the recruitment of nuclear receptor coactivators and effectively stimulate the transcription of their target genes. Different PPAR ligands may elicit distinct downstream biological effects due to unique conformational changes in the nuclear receptor.

**Figure 3:**
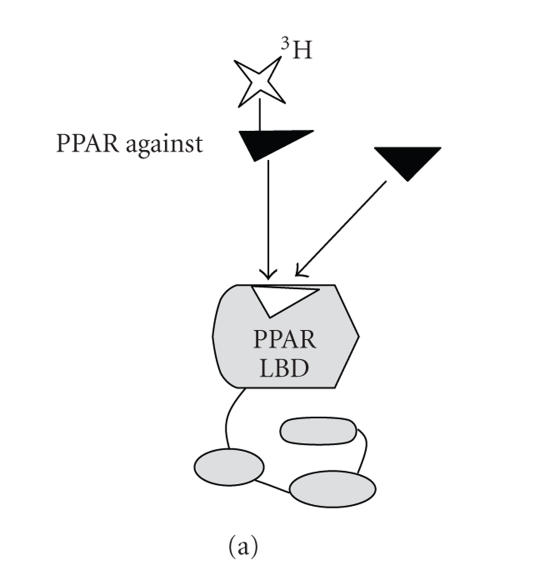

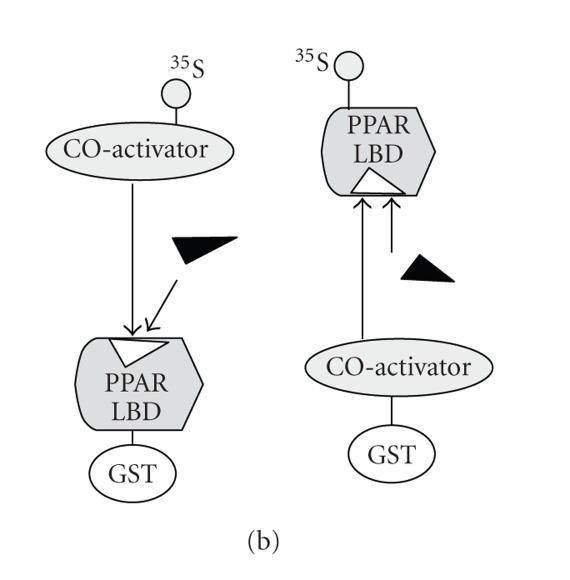

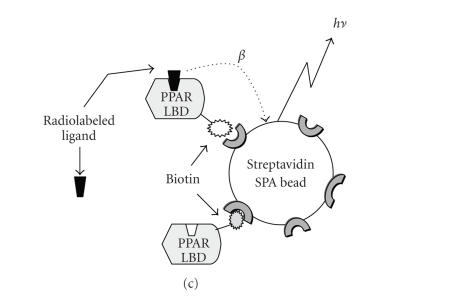

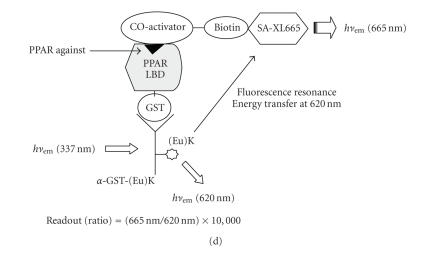

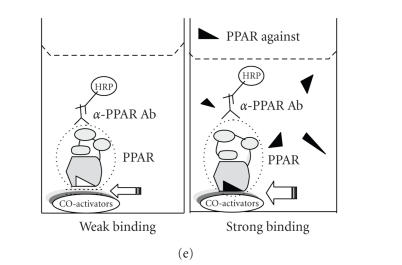

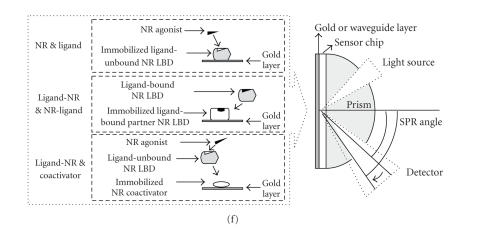
Various cell-free assays to discover PPAR modulator. A. Competition radioreceptor assays are performed by incubating recombinant PPAR protein and radioisotope-labeled ligand in the presence of competitor ligands. Bound ligands are separated from free forms by filtration. The amount of bound radioisotope-labeled ligand is determined by liquid scintillation counting. B. Coactivator-dependent receptor ligand assays (CARLAs) based on the recruitment of a coactivator due to a conformational change of specific ligand-bound PPAR. CARLA is carried out by incubating GST-PPAR and radioisotope-labeled coactivator with a ligand candidate or by incubating radioisotope-labeled PPAR and GST-coactivator with ligand candidates. The amount of ligand-bound PPAR-coactivator complex is determined by pull-down assay. C. In the scintillation proximity assay (SPA), the receptor-ligand complex is bound to the SPA bead through interaction between the biotinylated receptor and the streptavidin moiety located on the surface of the SPA bead. Because no separation step is required, SPA has benefits in its application to HTS. D. In the fluorescence resonance energy transfer (FRET)-based in vitro recruitment assay, GST-PPAR LBD proteins are indirectly linked to EU cryptate, (Eu)K, through (Eu)K-labeled anti-GST antibody, *α*-GST-(Eu)K. Purified recombinant coactivator is biotinylated, and is indirectly linked to XL665, which is produced only when there is a ligand-induced change in receptor conformation that results in binding to the coactivator. The extent of the FRET is measured as a ratio of 665 nm/620 nm X 10,000. E. A simple ELISA based on binding between PPAR and coactivators. The ligand unbound-PPAR weakly binds to the LXXLL motifs of coactivator, whereas ligand loaded-PPAR strongly binds to the LXXLL motifs of coactivator due to the conformational change of PPAR by specific agonists. This binding is detected by a specific anti-PPAR antibody, followed by horseradish peroxidase-conjugated secondary antibody. F. Schematic representation of SPR technology. One of the interacting partners is immobilized on a gold or waveguide layer of the sensor chip using the standard amine-coupling protocol. The other flows over the surface of the sensor chip, allowing interaction with the immobilized interacting partners. The interaction of immobilized partners with interacting molecules gives rise to an increase in mass. The refractive index and the angle of reflected light is thereby changed. As soon as the injection is stopped, the complex is washed with a washing buffer. The interacting molecules are dissociated from the immobilized interacting partner, resulting in a decrease in the signal due to a shift in the angle of the reflected light to its original position.

A cell-free competition radioreceptor assay using competitive interaction between a recombinant PPAR protein and a radioisotope-labeled ligand in the presence of competitor ligands has been reported previously (Figure 3(a)) [101, 102]. The coactivator-dependent receptor ligand assay (CARLA) has been reported as a cell-free assay based on the interaction between PPAR and the coactivator. CARLA is based on the recruitment of a transcriptional coactivator by changes in the conformation of ligand-bound PPAR (Figure 3(b)). In presence of PPAR ligands,^35^S-labeled SRC-1 has stronger interaction with GST-fused PPAR proteins immobilized on glutathione Sepharose beads. Autoradiograms and the quantification of the effect of candidates are dependent on the retention of SRC-1 by the GST-PPAR LBD [76]. The major advantage of CARLA is that it does not require radioactive labeling of candidate modulators, which makes it possible to screen a large number of compounds with this assay; this has simultaneous economic advantages in terms of materials and time.

Scintillation proximity has been developed as a tool for measuring the interaction between a receptor and a ligand. The scintillation proximity assay (SPA) bead is impregnated with a scintillant and coated with a capture molecule such as streptavidin. After preincubation of SPA beads and biotinylated PPAR LBD, radiolabeled ligands were added to a complex of SPA bead-PPAR LBD. Unbound free ligands were eliminated from the SPA-PPAR complex. When SPA-bead and radiolabeled ligands come into close proximity, radioactive counts are determined by the *β* emission from the radioisotope to be absorbed by the scintillant, which will then shift this energy to produce light (Figure 3(c)) [103, 104]. The advantages of the SPA are as follows: low cost, high sensitivity, high reliability, and simplicity, that is, no separation step is required. The simplicity of SPA is an important benefit in its application to HTS. However, unsuitability for kinetic determination and the limited number of useful isotopes were perceived as potential disadvantages.

Fluorescence has been considered as an analytical technique with which to study for the detection and quantitation of interacting molecules. There are several advantages to this technique, including high sensitivity, the relative ease of handling and disposal compared to radioactivity, and the diversity of available fluorophores. Thus, fluorescence resonance energy transfer (FRET) has been applied in the probing of molecular interactions [105]. As shown in Figure 3(d), GST-PPAR LBD was indirectly linked to Eu(K) through an anti-GST antibody, which was covalently linked to Eu(K). Coactivator was also indirectly linked to XL665 through a streptavidin (SA)-biotin adapter. The conformational change of PPAR by the PPAR agonist induces the recruitment of coactivator, and the interaction between PPAR and a coactivator will result in the close proximity of the fluorescence donor and acceptor. Consequently, the fluorescence donor (anti-GST-Eu(K)) is excited, and inputted energy will be transferred to the acceptor (streptavidin-XL665). Homogenous time-resolved fluorescence (HTRF) energy transfer technology takes advantage of fluorescence, as well as the homogenous and time-resolved detection mode. These specificities of HTRF enable it to overcome most of the drawbacks encountered in FRET [106].

Previous cell-free methods with which to screen PPAR agonists have used isotope or fluorescence labeling agonists or proteins. We established very simple ELISA systems based on the ligand-dependent binding between PPAR and coactivators. In brief, the purified recombinant LXXLL motif of coactivators was applied into a 96-well plate, and *E. coli* lysates containing recombinant PPAR proteins were then added with candidate PPAR agonists. The complex consisting of PPAR and coactivator was then identified with the anti-PPAR antibody (Figure 3(e)). Major advantages of this simple method are its simplicity and its low cost, as these systems do not require any labeling of candidate modulators and proteins. This makes it possible to screen a large number of compounds, with simultaneous economic advantages in terms of materials and time. On the other hand, this method has relatively low sensitivity and has to use a suitable anti-PPAR antibody [107–109].

In the 1980s, surface plasmon resonance (SPR) and related techniques that exploited evanescent waves were applied for the study of biological and chemical interactions. SPR technology has also been successfully employed to study the interactions between ligands and nuclear receptors [110–112], the effects of ligand-binding on nuclear receptor dimerization [113], and ligand screening based on interactions between ligand-bound nuclear receptors and coactivators [114, 115] (Figure 3(f)). In the interaction analysis between PPAR LBD and ligand, PPAR LBD is immobilized on the sensor chip by a standard primary amine-coupling reaction, and the ligand is injected over the immobilized PPAR LBD. In the investigation of ligand binding on receptor dimerization, the partner nuclear receptor is immobilized on the sensor chip by the standard amine-coupling reaction, and the nuclear receptor pre-incubated with its ligand is injected over the immobilized partner nuclear receptor. In ligand screening based on interactions between ligand-bound nuclear receptors and coactivator, the coactivator or LXXLL peptide is immobilized on the sensor using same methods, and the nuclear receptor that was preincubated with a candidate chemical is injected over the immobilized coactivator peptide. The association (*k_a_*) and dissociation (*k_d_*) rate constants and the dissociation equilibrium constants (*k*_D_s) for the bindings were determined using the Biacore biosensor. The binding responses in resonance units (RUs) were continuously recorded, and were presented graphically as a function of time. SPR technology has the advantages in that it requires no labeling, can be performed in real-time, and utilizes noninvasive measurements.

## 6. SUMMARY

Obesity mainly reflects an increased adipose cell size, an increased adipocyte cell number, and an imbalance between energy intake and energy expenditure. Excess body fat is associated with metabolic disorders. As a molecular drug target for metabolic disorders, the activation of PPAR by specific ligands has many beneficial clinical effects in the improvement of glucose and lipid homeostasis. The PPARs mainly consist of three subtypes (PPAR*α*, PPAR*γ*, and PPAR*δ*), and all three PPAR isoforms possess similar structural and functional features involving glucose metabolism, lipid metabolism, and energy balance. PPARs directly modulate gene expression upon binding to specific ligands transferred into PPAR via iLBP-mediated translocation. PPAR agonists play an important role in therapeutic aspects of metabolic disorders, whereas undesired effects for the existing PPAR agonists prescribed as therapeutic agents have been reported. To discover new PPAR modulators with more efficacious effects and more safety against undesired side effects, a novel PPAR antagonist or the combination of agonists such as PPAR*α*/*γ* dual agonist, PPAR*γ*/*δ* dual agonist, PPAR*α*/*δ* dual agonist, and PPARpan (PPAR*α*/*γ*/*δ*) agonist has been applied to activate each receptor subtype and selective PPAR*γ* modulators (SPPAR*γ*Ms). In addition, various technologies have been developed in attempts to discover PPAR modulators as therapeutic agents for the treatment of metabolic disorders. Because cell-based assays have more physiological relevance, the transactivation assay, chimeric receptor transactivation, and yeast two-hybrid methods have also been used. Since cell-free assays are based on direct interaction between PPAR and their specific ligands, a new concept for competing radioreceptor assays has been developed by making the best use of competitive interactions between recombinant PPAR protein and radioisope-labeled ligands. Later, cell-free assays (CARLA and SPA) were developed based on conformational changes in PPARs caused by their ligands, and the simplicity of SPA permitted application to high-throughput screening (HTS). Radioisotope-free assays like FRET (HTRF), ELISA, and SPR methods are relatively simple in terms of handling and disposal. Thus, HTRF and SPR assays can be applied to a homogenous and time-resolved detection mode.

Interestingly, prior to the discovery of the PPAR*α*, it was reported that Wy-14,643, a well-known synthetic agonist of PPAR*α*, decreased serum cholesterol and triglyceride levels in mice [116]. TZD derivatives, well-known synthetic agonists of PPAR*γ*, were reported as antidiabetes agents prior to the discovery of the PPAR*γ* [117, 118]. An antidiabetes agent, pioglitazone, a TZD derivative, has recently been shown to bind specifically to a protein named mitoNEET [119]. These studies of PPAR, PPAR modulators, and technologies to discover PPAR modulators will elicit the development of drugs with more efficacious effects and more safety for the treatment of metabolic disorders.
